# Genes associated with polymorphic variants predicting lung function are differentially expressed during human lung development

**DOI:** 10.1186/s12931-016-0410-z

**Published:** 2016-07-29

**Authors:** S. Miller, E. Melén, S. K. Merid, I. P. Hall, I. Sayers

**Affiliations:** 1 Division of Respiratory Medicine, Queen’s Medical Centre, University of Nottingham, Nottingham, NG7 2UH UK; 2 Institute of Environmental Medicine, Karolinska Institutet, Stockholm, Sweden; 3 Sachs’ Children’s Hospital, Stockholm, Sweden

**Keywords:** Lung function, Development, Expression, Genetics, TMEM163, CDC123

## Abstract

**Background:**

Recent meta-analyses of genome-wide association studies have identified single nucleotide polymorphisms (SNPs) within/near 54 genes associated with lung function measures. Current understanding of the contribution of these genes to human lung development is limited. We set out to further define i) the expression profile of these genes during human lung development using a unique set of resources to examine both mRNA and protein expression and ii) the link between key polymorphisms and genes using expression quantitative trait loci (eQTL) approaches.

**Methods:**

The mRNA expression profile of lung function associated genes across pseudoglandular and canalicular stages of lung development were determined using expression array data of 38 human fetal lungs. eQTLs were investigated for selected genes using blood cell and lung tissue data. Immunohistochemistry of the top 5 candidates was performed in a panel of 24 fetal lung samples.

**Results:**

Twenty-nine lung function associated genes were differentially expressed during lung development at the mRNA level. The greatest magnitude of effect was observed for 5 genes; *TMEM163, FAM13A* and *HHIP* which had increasing expression and *CDC123* and *PTCH1* with decreased expression across developmental stages. Focussed eQTL analyses investigating SNPs in these five loci identified several cis-eQTL’s. Protein expression of TMEM163 increased and CDC123 decreased with fetal lung age in agreement with mRNA data. Protein expression in FAM13A, HHIP and PTCH1 remained relatively constant throughout lung development.

**Conclusions:**

We have identified that > 50 % of lung function associated genes show evidence of differential expression during lung development and we show that in particular TMEM163 and CDC123 are differentially expressed at both the mRNA and protein levels. Our data provides a systematic evaluation of lung function associated genes in this context and offers some insight into the potential role of several of these genes in contributing to human lung development.

**Electronic supplementary material:**

The online version of this article (doi:10.1186/s12931-016-0410-z) contains supplementary material, which is available to authorized users.

## Background

Determinants of lung function and adult lung diseases, such as Chronic Obstructive Pulmonary Disease (COPD) may have early origins [1–4]. Current knowledge of human lung development is based on evidence provided by anatomical dissections and histological staging. Historically, lung development has been divided into 5 stages of growth due to the presence of select morphological features, Embryonic (26 days post conception −5 weeks), Pseudoglandular (5–16 weeks), Canalicular (16–26 weeks), Saccular (26 weeks-birth) and Alveolar (birth to 6 months). Previous work by Kho et al. sought to further our understanding of human lung development and consider differences in expression at the molecular level [5]. The authors investigated the hypothesis that the temporal changes in gene expression regulated the morphological transformation of early cells to a fully functional differentiated organ. Profiling the gene expression signatures in a panel of human fetal lungs identified differential gene expression during organ development and revealed the presence of several molecular stages. The early pseudoglandular stage was enriched for genes involved in chromosomal organisation processes associated with mitosis. The late pseudoglandular stage was enriched for genes related to surfactant function-gas exchange and immunological-MHC class II attributes providing evidence of at least a second distinct molecular phase of development [5].

Recently, five Genome-Wide Association Study (GWAS) meta-analyses have been completed that identified single nucleotide polymorphisms (SNPs) within or near 54 genes associated with spirometry measures including; Forced expiratory volume in 1 s (FEV_1_), forced vital capacity (FVC) and the ratio of FEV_1_/FVC [6–10]. These measures of lung function are commonly used to define respiratory diseases such as COPD. To date, no studies have explored the potential role of these genes in human lung development by expression profiling this set of genes in human fetal lung. We hypothesised that these SNPs identified by GWAS tag lung function genes which may contribute to the lung developmental signatures described [5]. This underlying hypothesis is supported by data from large meta-analyses of these lung function GWAS as association signals were observed both in childhood and adult cohorts suggesting early origins of effects [6]. We therefore set out to identify whether the genes potentially underlying these associations identified in lung function GWAS were i) differentially expressed across stages of human lung development using mRNA analyses; ii) if we could identify expression quantitative trait loci (eQTL) in these genomic regions contributing to the levels of these specific genes underlying mechanisms and (iii) if the alteration in mRNA is translated to altered protein levels for selected candidates.

## Methods

### Selection of genes and gene expression array analysis

We identified genes to study by focussing on studies using GWAS meta-analysis approaches and taking the gene(s) in each loci that were reported as the likely candidate in these loci by the authors based on available evidence [6–10]. This approach generated a list of 54 genes for analyses (Additional file 1. Table S1). Publically available Affymetrix U133 Plus 2 expression array data of 38 fetal lung samples with gestational age 7–22 weeks (Gene Expression Omnibus (GEO) dataset, GSE14334 [5, 11]) were used to identify differential expression during lung development for the 54 genes. A total of 190 probes were interrogated with some genes represented by a single probe, while others had up to 12 probes (Additional file 2: Table S2). Briefly, differential gene expression analysis relative to gestational age was performed using a linear regression model [11]. Multiple testing was corrected for using the Benjamini and Hochberg method. Adjusted *p* values of < 0.05 were used to show a significantly differential gene expression pattern over the gestational ages studied.

### *In silico* linkage disequilibrium and eQTL analyses

The top 5 differentially expressed genes were taken forward for *in silico* investigation. We focussed our analyses to the pivotal SNPs reported in the original manuscript and all other SNPs in linkage disequilibrium with these SNP (r^2^ > 0.8), identified using Haploreg version 2 which is data based on 1000 Genomes Phase I individuals (db SNP 137). These SNPs were subsequently assessed for cis- and trans-eQTL’s in (i) blood using the online blood eQTL browser (http://genenetwork.nl/bloodeqtlbrowser/) detected at a false discovery rate (FDR) of 5 % [12] and in (ii) 1111 lung tissue resections from a study by Hao et al. [13] at a FDR of 10 %.

### Immunohistochemistry of selected genes

The top 5 differentially expressed genes were taken forward for immunohistochemical protein analysis using 24 formalin-fixed paraffin-embedded fetal lung samples (19 days–19 weeks gestation). Samples collected for immunohistochemical staining were consented for in accordance with national bio banking procedures and the UK human tissue act (2004). The human embryonic and fetal material was provided by the Joint MRC/Wellcome Trust (grant # 099175/Z/12/Z) Human Developmental Biology Resource (www.hdbr.org). Samples were collected at diverse stages of development, specifically 19, 21, 22 and 23 days and 9, 10, 11, 12, 13, 14, 15, 16, 17 and 19 weeks post-conception. For all samples, 4 μm whole tissue sections on glass slides were de-paraffinised in Histo-clear (National Diagnostics, Dublin, Ireland) and hydrated using decreasing concentrations of ethanol. Antigen retrieval was performed in a steamer for 20 min in sodium citrate buffer (pH 6.0), followed by an endogenous peroxidise block for 5 min (Dako, Cambs, UK). Sections were incubated for 1 h at room temperature with primary antibodies for CDC123 (1 in 500, HPA037830, Sigma, Dorset, UK), TMEM163 (1 in 100, HPA007224, Sigma, Dorset, UK), HHIP (1 in 100, H00064399-M01, Novus Biologicals Europe, Cambridge, UK), PTCH1 (1 in 100, 21130002, Novus Biologicals Europe, Cambridge, UK) and FAM13A (1 in 500, HPA038109, Sigma, Dorset, UK). Isotype controls were included where sections were treated with normal rabbit or mouse IgG as a matched isotype control (Invitrogen/Life Technologies, Paisley, UK). The Dako Chemate Envision Detection Kit (Dako) with DAB chromogen was used for detection. Sections were subsequently counterstained with Mayer’s Haematoxylin (Sigma- Aldrich, Dorset, UK), dehydrated and a coverslip mounted using Vectamount (Vector Laboratories, Peterborough, UK). Antibodies were chosen due to their specific staining patterns in a large bank of human tissues in the protein atlas (www.proteinatlas.org). Normal control human lung was used as a positive control for CDC123 and TMEM163 staining, Tonsil for HHIP and PTCH1 and Bronchus for FAM13A (Additional file 3: Figure S1A, C, E, G, I, respectively). Control tissues were provided by the Nottingham Health Science Biobank (Nottingham, UK) under ethical approval (08/H0407/1). Isotype controls were also performed (Additional file 3: Figure S1B, D, F, H, J). Results were visualised using an Olympus BX14 light microscope.

## Results

### Lung function genes are expressed in human fetal lung and show differential expression

To identify whether the 54 lung function genes (Additional file 1: Table S1) were expressed during normal human lung development, we utilised the gene expression array data of 38 lung samples across the Pseudoglandular (gestational age, 7–16 weeks) and Canalicular (17–26 weeks) stages. All 54 genes were identified as expressed in the fetal lung (average probe expressions from 3.08 to 10.66) (Additional file 2: Table S2) and 29/54 genes were found to have some evidence of differentially expressed probes across the Pseudoglandular and Canalicular stages of development (Additional file 4: Table S3).

### Identification of genes with greatest magnitude of differential expression

Significant probes in lung function associated genes from the expression profiles of 38 fetal lung samples (adjusted *p* value < 0.05) were ordered by Beta coefficient to highlight the greatest magnitude of difference in expression (mean change in gene expression per day) across the developmental stages (Tables 1 and 2).

**Table 1:** Top differentially expressed gene probes showing increase in expression with increasing fetal lung age

Gene	Locus	Probe	Beta coefficient
*TMEM163*	2p21.3	223503_at	0.0328382
*TMEM163*	2p21.3	1552626_a_at	0.0219712
*FAM13A*	4q22.1	232628_at	0.0154821
*AGER*	6p21.32	210081_at	0.0150551
*HHIP*	4q31.21	1556037_s_at	0.0138319
*C10orf11*	10q22.2	240772_at	0.0134878
*HHIP*	4q31.21	237466_s_at	0.0119547
*FAM13A*	4q22.1	243020_at	0.0117218
*NPNT*	4q24	225911_at	0.0113676
*PDE4D*	5q12.1	236610_at	0.0104328

All probes in adjusted *p* value of <0.05. Probe = Affymetrix U133 Plus 2 array probe ID. Beta coefficient corresponds to the mean change in gene expression per day during the studied period (7–22 weeks of gestational age)

**Table 2:** Top differentially expressed gene probes showing decrease in expression with increasing fetal lung age

Gene	Locus	Probe	Beta coefficient
*PRDM11*	11p11.2	229687_s_at	−0.0145780
*PTCH1*	9q22.32	209815_at	−0.0123042
*GSTCD*	4q24	235387_at	−0.0115404
*PTCH1*	9q22.32	209816_at	−0.0109530
*MTHFD1L*	6q25.1	225520_at	−0.0103665
*CDC123*	10p13	201725_at	−0.0096350
*CDC123*	10p13	223100_s_at	−0.0090160
*WWOX*	16q23.1	223868_s_at	−0.0082566
*BCL2*	18q21.33	207004_at	−0.0078140
*PRDM11*	11p11.2	229688_at	−0.0076778

All probes in adjusted *p* value of <0.05. Probe = Affymetrix U133 Plus 2 array probe ID. Beta coefficient corresponds to the mean change in gene expression per day during the studied period (7–22 weeks of gestational age)

### Identification of genes for further study

**Fig. 1:**
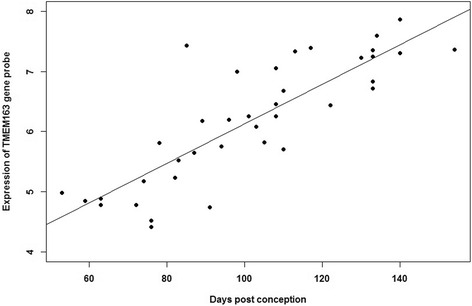
*TMEM163* mRNA expression in human lung across Pseudoglandular and Canalicular stages of development. Expression of *TMEM163* gene probe 230135_at showed an increase in mRNA expression with increasing fetal lung age. Affymetrix U133 Plus 2 expression array probe data of 38 fetal lung samples with gestational age 7–22 weeks

**Fig. 2:**
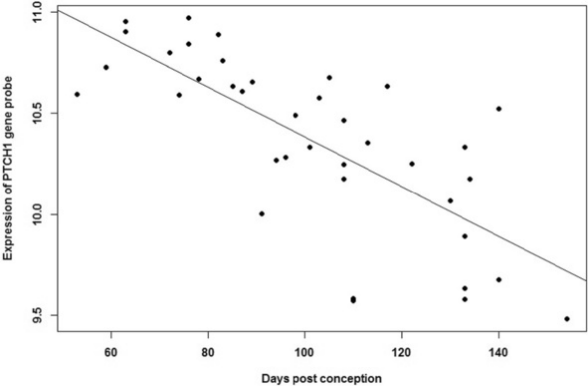
*PTCH1* mRNA expression in human lung across Pseudoglandular and Canalicular stages of development. Expression of *PTCH1* gene probe 202973_x_at showed a decrease in mRNA expression with increasing fetal lung age. Affymetrix U133 Plus 2 expression array probe data of 38 fetal lung samples with gestational age 7–22 weeks

Genes with at least 2 of the most significantly differentially expressed probes, a consistent direction of effect and the majority of tested probes showing this effect were taken forward for further analysis. These included Hedgehog interacting protein (*HHIP*), Transmembrane protein 163 (*TMEM163*), Family with sequence similarity 13, member A (*FAM13A*), Patched1 (*PTCH1*) and Cell division cycle 123 (*CDC123*). *HHIP*, *TMEM163* and *FAM13A* showed an increased mRNA expression with fetal lung age (Table 1), whilst *PTCH1* and *CDC123* showed a significant decrease in expression (Table 2). For visualisation, the most significant probe results for each of these selected genes were plotted (Figs. 1 and 2, Additional file 5: Figure S2, Additional file 6: Figure S3 and Additional file 7: Figure S4). Expression intensities were plotted against gestational age of the developing lung samples.

### eQTL analyses of SNPs associated with lung function measures in the 5 gene loci identified for further study

Nine SNPs within our 5 gene regions of interest have previously been associated with lung function at genome wide significance levels (*P* < 10^−8^) (Table 3).

**Table 3:** SNPs within 5 top differentially expressed genes associated with lung function

Gene	Associated SNP	Locus	Lung function measure	Coded Allele	Direction of effect	Reference
*CDC123*	rs7068966	10p13	FEV_1_	T	+	Soler-Artigas et al. [9]
			FEV_1_/FVC	T	+	
*PTCH1*	rs16909898	9q22.32	FEV_1_/FVC	A	+	Hancock et al. [7]
	rs10512249		FEV_1_/FVC	A	−	
*TMEM163*	rs1942055	2p21.3	FVC	G	−	Loth et al. [10]
*FAM13A*	rs2869967	4q22.1	FEV_1_/FVC	T	+	Hancock et al. [7]
	rs6830970		FEV_1_/FVC	A	+	
*HHIP*	rs1980057	4q31.21	FEV_1_/FVC	T	+	Hancock et al. [7]
	rs1032295		FEV_1_/FVC	T	−	
	rs12504628		FEV_1_	T	−	Repapi et al. [6]
			FEV_1_/FVC	T	−	

To investigate whether lung function associated SNPs within the top 5 differentially expressed genes during lung development had evidence of regulated expression, *in silico* analyses were performed to identify (i) SNPs in Linkage Disequilibrium (LD) with the SNPs of interest (R^2^ > 0.8) and (ii) expression quantitative trait loci (eQTL) for the SNP of interest and those in LD. Apart from *HHIP* SNP rs1032295, all lung function associated SNPs had SNPs in LD (Additional file 8: Table S4). eQTL were identified for SNPs in LD with lung function associated SNPs in *PTCH1, TMEM163* and *FAM13A* using the blood cell dataset (Additional file 8: Table S4). 12 cis-eQTLs were identified for SNPs in LD with rs16909898 and rs10512249 (*PTCH1*) which potentially regulate the expression levels of *PTCH1* mRNA. Additionally 2 trans-eQTLs were identified for lung function associated SNP rs16909898 and rs10512249 (*PTCH1*), both regulating the expression of *NEFH* (Neurofilament, Heavy Polypeptide, chromosome 22). 3 cis-eQTLs were identified for SNPs in LD with *TMEM163* SNP rs1942055, regulating the expression of either *MGAT5* (encoding a member of the glycosyltransferase family) or *CCNT2* (encoding Cyclin T2). eQTL were only identified for SNPs in LD with *HHIP* using the human lung dataset (Additional file 9: Table S5). Both rs1980057 and rs12504628 shared 2 SNPs in LD (rs12509311 and rs13141641) and were cis-eQTL’s for *HHIP* (Additional file 9: Table S5). eQTLs in both Blood and Lung cohorts studied were not identified for SNPs in LD with *CDC123* SNP rs7068966.

### Analyses of differential protein expression across lung developmental stages

**Fig. 3:**
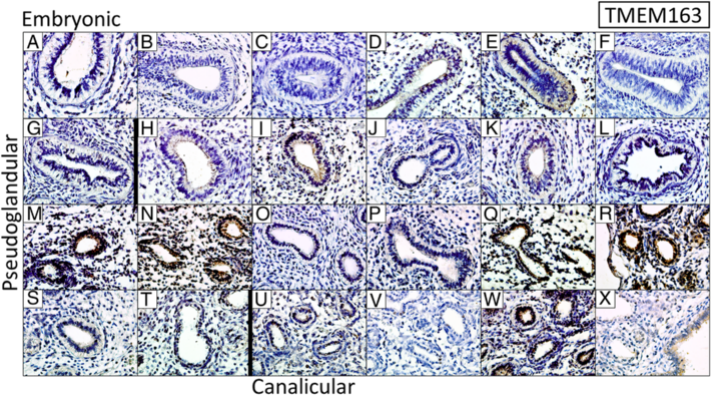
Immunohistochemistry for TMEM163 in 24 fetal lung samples. Protein expression increases across Pseudoglandular and Canalicular stages of human fetal lung development. **a**–**g** embryonic stage, **h**–**t** Pseudoglandular stage and **u**–**x** Canalicular stage. An isotype control (not shown) gave no background staining. ×40 Magnification

**Fig. 4:**
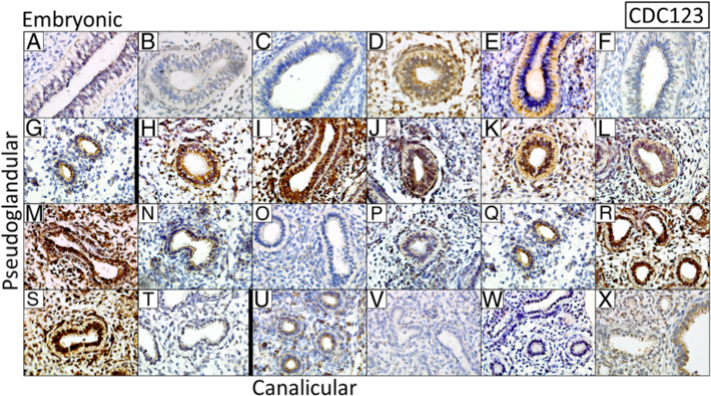
Immunohistochemistry for CDC123 in 24 fetal lung samples. Protein expression decreases across Pseudoglandular and Canalicular stages of human fetal lung development. **a**–**g** embryonic stage, **h**-**t** Pseudoglandular stage and **u**-**x** Canalicular stage. An isotype control (not shown) gave no background staining. ×40 Magnification

Protein expression was assessed for TMEM163, CDC123, HHIP, PTCH1 and FAM13A using immunohistochemistry in 24 formalin fixed paraffin embedded fetal lung samples. We found some variability in the levels of HHIP, PTCH1 and FAM13A expression between lung samples however; consistent effects were seen for TMEM163 and CDC123. Gene expression array probes for *TMEM163* showed the greatest magnitude of effect across the pseudoglandular and canalicular stages of lung development (Table 1 and Fig. 1). In the immunohistochemical assessment, TMEM163 protein was either not present or at very low levels in the embryonic fetal lung samples, 4/13 pseudoglandular stage lungs had strong immunopositivity, whilst 2/4 canalicular stage lungs had strong/moderate positive protein expression (Fig. 3). Whilst the earliest 12 fetal lungs (7 embryonic and 5 pseudoglandular) were negative for TMEM163, of the latest 12 (8 pseudoglandular and 4 canalicular), half were strongly immunopositive. CDC123 protein expression was found to decrease between Pseudoglandular and Canalicular stages of lung development (Fig. 4). 4/7 fetal lungs at the embryonic stage were immunopositive for CDC123, 11/13 lung samples at the Pseudoglandular stage of development had strong/moderate immunopositivity for CDC123 whilst 2/4 lung samples at the Canalicular stage had low CDC123 expression. Overall, CDC123 decreased in expression from the pseudoglandular to canalicular stages. More variable protein expression was observed for PTCH1, FAM13A and HHIP (Additional file 10: Figures S5, Additional file 11: Figure S6 and Additional file 12: Figure S7, respectively). Whilst PTCH1 mRNA expression was found to decrease with increasing fetal age, the immunohistochemical analyses showed that, apart from the 3 earliest embryonic lungs, the majority of fetal lungs had strong or moderate staining for the PTCH1 protein (Additional file 10: Figure S5). Few fetal lung samples showed the presence of FAM13A protein expression; the majority of samples had low level expression or were negative (Additional file 11: Figure S6). HHIP had variable protein expression throughout lung development, with either negative or moderate staining identified (Additional file 12: Figure S7).

## Discussion

Recent meta-analyses of genome-wide association studies have identified genetic associations which contribute to determination of lung function. We hypothesised that the genes underlying these associations may contribute to lung development potentially through differential expression. To specifically test this hypothesis we expression profiled lung function associated gene mRNA levels in human fetal lung samples and used eQTL analyses to link associated SNPs to the genes of interest, followed by expression profiling at the protein level in human fetal tissue. We found that all 54 genes evaluated had measurable mRNA expression in human fetal lung and 29/54 genes showed evidence of differential expression across the Pseudoglandular and Canalicular stages of lung development. We identified cis-eQTLs in Blood for SNPs in LD with lung function associated SNPs in *PTCH1*, *TMEM163* and *FAM13A* and cis-eQTLs in *HHIP* in the lung. Although no cis-eQTL were identified for SNPs in LD with *CDC123* or the sentinel SNP associated with lung function and *CDC123*, we still sought to explore the protein expression of this highly differentially expressed candidate. The rationale for this inclusion was that while eQTL analyses can be useful for functional translation of SNP effects these analyses are tissue, cell and context specific [14]. Therefore based on our analyses of blood and lung eQTL data we cannot exclude that eQTL mechanisms may exist for *CDC123*. Overall, these data provided evidence that the associated SNPs in the relevant loci showed some evidence of regulating the genes that were differentially expressed in our initial analyses and selected for further study.

Exploring the top 5 candidates further (based on magnitude of effect and reproducibility across probes), we found that TMEM163 protein expression generally increased with fetal age and CDC123 protein expression decreased in agreement with the mRNA data. Of the 5 candidates taken forward for the immunohistochemical study in fetal lungs we found both CDC123 expression (decreased) and TMEM163 (increased) provided the greatest supporting evidence at the protein level, whilst the mRNA and protein level data for PTCH1, FAM13A and HHIP poorly correlated.

In our first set of analyses we identified that 29/54 (>53 %) genes identified in lung function associated loci in GWAS were differentially expressed across human lung development. This is nearly double the percentage of probes differentially expressed during the analysed period of lung development (28 %, see [11]). This enrichment is striking and adds supporting evidence that genetic determinants of lung function may have early origins for a large proportion of loci. While differential expression is not conclusive evidence of a significant biological role, it is also important to note that many of the lung function genes have prior evidence for a role in lung development from both mouse and human studies; e.g., *HHIP, PTCH1* [15, 16].

Interestingly, these genes were in our 5 top candidates and are part of the Sonic hedgehog (SHH) signalling pathway. *PTCH1* encodes the receptor potentiating signalling, whilst expression of *HHIP* represses the transcription of HH target genes. We found expression of *PTCH1* mRNA and protein expression was generally high throughout lung development, with probe expression intensities > 9 and strong immunopositivity in the majority of fetal lungs which is consistent with other studies in human fetal lung [15]. High protein levels of PTCH1 have been seen in nearly all tissues in adulthood including pneumocytes, macrophages and epithelium of the lung (www.proteinatlas.org). Thus, the evidence suggests that PTCH1 not only has a role in lung development but may also play an important role in determining lung function later in adult life [17]. The second SHH pathway candidate investigated was HHIP, an important morphogen in a variety of developmental processes during embryonic development and SNPs near this gene have previously been associated with risk of COPD [18, 19]. In 2013, S. Collins et al.*,* summarised that polymorphisms in HHIP affect fetal, childhood and adult lung function [20]. HHIP is known to have a role in lung development through fibroblast growth factor 10 (FGF10) and its control of lung branching [21].

Of the most robust findings; i.e., where mRNA differential expression was supported by differential protein expression in the same direction; CDC123 expression (decreased) and TMEM163 (increased) warrant further study. CDC123 is thought to be required for translation initiation and thus could facilitate the biogenesis of the eukaryotic initiation factor 2 (eIF2) and is also potentially involved in protein modifications [22, 23]. At present, there is evidence that TMEM163 (SV13) is a zinc finger binding protein involved in vesicular transport [24] and more recent work has shown TMEM163’s modulation of cellular zinc levels alongside TRPML1 [25].

A separate candidate was *FAM13A*, which has previously been associated with other lung conditions, e.g., idiopathic pulmonary fibrosis and COPD. It has been hypothesised that FAM13A has a role in Rho GTPase signalling pathways [26] and most recently Jin *et al.* has identified the regulation of nuclear–cytoplasmic shuttling of FAM13A by B56-containing PP2As and Akt [27]. Additionally, they identified that FAM13A had the ability to activate the Wnt pathway. However, on production of *FAM13A-/-* mutant mice, they were found to be viable and healthy, showing FAM13A was not essential for embryonic development and physiological functions.

Working with fetal tissues has both strengths and limitations. Key strengths of the current study are the unique use of human fetal samples across gestational ages for both mRNA and protein analyses with associated translational potential. However, it was not possible to use the same sample for both mRNA and protein analyses which introduces sample variation potentially confounding correlations between mRNA and protein expression. Correlation between mRNA and protein throughout lung development may not be present due to the complexities of post-transcriptional regulation. Other limitations of the study were the availability of only a small number of samples for immunohistochemistry at the Canalicular stage of lung development and that the protein analyses were based on immunohistochemistry rather than a more quantitative method e.g., Western blotting, prohibiting quantitative or semi-quantitative analyses.

## Conclusion

This is the first study to comprehensively investigate the mRNA expression of the genes closest to association signals seen from GWAS of lung function during human lung development with focussed analyses for 5 candidates at both the RNA and protein level. The data presented demonstrate that >50 % of these genes show some evidence of differential expression during normal human lung development. We have provided evidence that TMEM163 (a transmembrane protein) and CDC123 (a cell cycle control protein) are differentially expressed at both the mRNA and protein level during lung development. These candidates now warrant further investigation as they may play an important role in determining lung function later in adult life.

## Abbreviations

SNP, single nucleotide polymorphism; eQTL, expression quantitative trait loci; COPD, chronic obstructive pulmonary disease; GWAS, genome-wide association study; FEV_1_, forced expiratory volume in 1 second; FVC, forced vital capacity; GEO, gene expression omnibus; LD, linkage disequilibrium

## Additional files

Additional file 1: Table S1. SNPs within/near 54 previously identified genes associated with lung function measures FEV_1_, FVC and FEV_1_/FVC. (XLSX 21 kb) Additional file 2: Table S2. Expression array probes and results annotated to 54 genes associated with lung function. Probe ID = Affymetrix U133 Plus 2 array probe ID, LogFC = log fold change, AveExpr = average expression throughout development, t = t-statistic describing differential expression, adj.P.Value = Adjusted p value controlling for false discovery rate. Beta coefficient corresponds to the mean change in gene expression per day during the studied period (7–22 weeks of gestational age). (XLSX 37 kb) Additional file 3: Figure S1. Positive and isotype control immunohistochemical staining for CDC123, TMEM163, HHIP, PTCH1 and FAM13A proteins. Lung tissue was immunopositive for CDC123 (A) and TMEM163 (C), Tonsil tissue was immunopositive for HHIP (E) and PTCH1 (G) and Bronchus was immunopositive for FAM13A (I). All isotype controls were negative (B, D, F, H and J). (JPG 19349 kb) Additional file 4: Table S3. Lung function associated genes with significantly different expression during lung development. Affymetrix U133 Plus 2 array probe ID. Beta coefficient corresponds to the mean change in gene expression per day during the studied period (7-22 weeks of gestational age). (DOCX 22 kb) Additional file 5: Figures S2. FAM13A mRNA expression in human lung across Pseudoglandular and Canalicular stages of development. Expression of FAM13A gene probe 201725_at showed an increase in mRNA expression with increasing fetal lung age. (JPG 43 kb) Additional file 6: Figures S3. HHIP mRNA expression in human lung across Pseudoglandular and Canalicular stages of development. Expression of HHIP gene probe 209815_at showed an increase in mRNA expression with increasing fetal lung age. (JPG 43 kb) Additional file 7: Figures S4. CDC123 mRNA expression in human lung across Pseudoglandular and Canalicular stages of development. Expression of CDC123 gene probe 223503_at showed a decrease in mRNA expression with increasing fetal lung age. (JPG 43 kb) Additional file 8: Table S4. Evidence of regulated expression (by LD and Blood eQTL) in 5 top differentially expressed genes associated with lung function. SNP = single nucleotide polymorphism, LD = linkage disequilibrium, Chr = chromosome, FDR = false discovery rate, eQTL = expression quantitative loci. (XLSX 28 kb) Additional file 9: Table S5. Evidence of regulated expression (by LD and Lung eQTL) in 5 top differentially expressed genes associated with lung function. (XLSX 33 kb) Additional file 10: Figures S5. Immunohistochemistry for PTCH1 in 24 fetal lung samples. The majority of fetal lungs showed strong or moderate immunopositivity for the PTCH1 protein. (A–G) embryonic stage, (H–T) Pseudoglandular stage and (U–X) Canalicular stage. An isotype control (not shown) gave no background staining. x40 Magnification. (TIF 3594 kb) Additional file 11: Figures S6. Immunohistochemistry for FAM13A in 24 fetal lung samples. Fetal lung samples showed either low level or negative protein expression for FAM13A. (A–G) embryonic stage, (H–T) Pseudoglandular stage and (U–X) Canalicular stage. An isotype control (not shown) gave no background staining. x40 Magnification. (TIF 3390 kb) Additional file 12: Figures S7. Immunohistochemistry for HHIP in 24 fetal lung samples. HHIP protein expression was either not present or moderate throughout lung development. (A–G) embryonic stage, (H–T) Pseudoglandular stage and (U–X) Canalicular stage. An isotype control (not shown) gave no background staining. x40 Magnification. (TIF 3261 kb)
