# Supplementary figures and images for: Genes associated with polymorphic variants predicting lung function are differentially expressed during human lung development
Source: Respir Res. 2016 Jul 29;17:95. doi: 10.1186/s12931-016-0410-z (PMC4966770; doi:10.1186/s12931-016-0410-z)

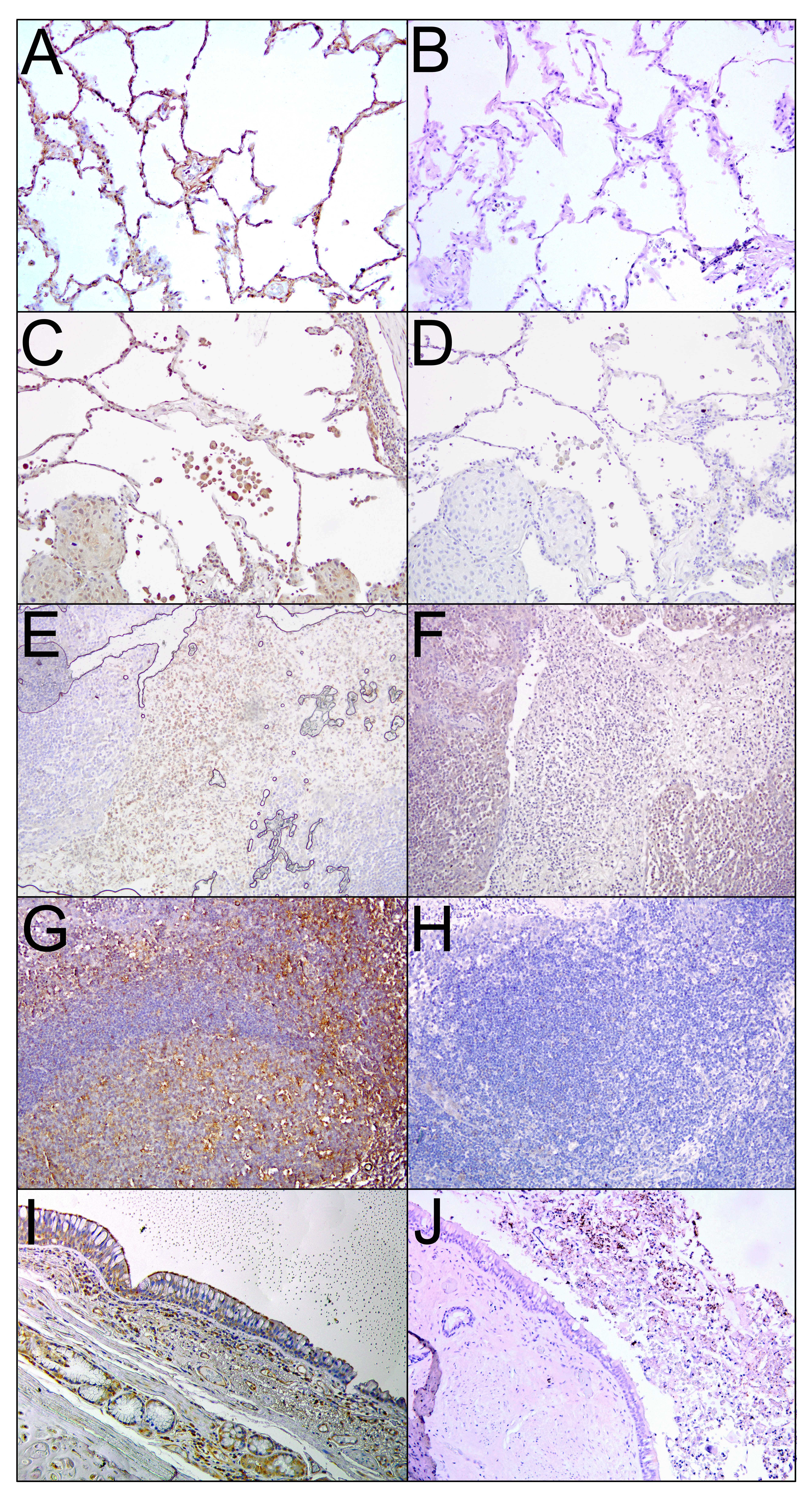

Supplement: Additional file 3: Figure S1. — Positive and isotype control immunohistochemical staining for CDC123, TMEM163, HHIP, PTCH1 and FAM13A proteins. Lung tissue was immunopositive for CDC123 (A) and TMEM163 (C), Tonsil tissue was immunopositive for HHIP (E) and PTCH1 (G) and Bronchus was immunopositive for FAM13A (I). All isotype controls were negative (B, D, F, H and J). (JPG 19349 kb) [file 12931_2016_410_MOESM3_ESM.jpg]

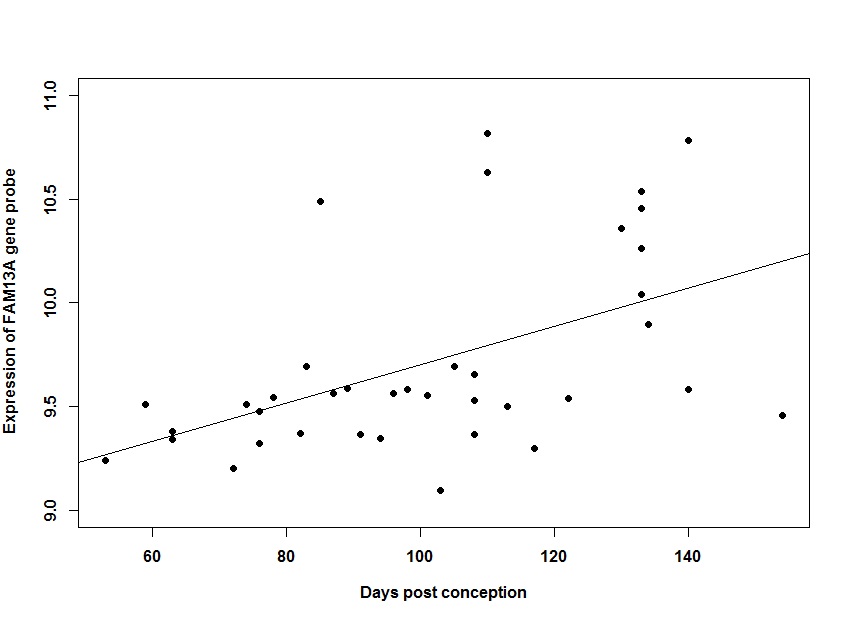

Supplement: Additional file 5: Figures S2. — FAM13A mRNA expression in human lung across Pseudoglandular and Canalicular stages of development. Expression of FAM13A gene probe 201725_at showed an increase in mRNA expression with increasing fetal lung age. (JPG 43 kb) [file 12931_2016_410_MOESM5_ESM.jpg]

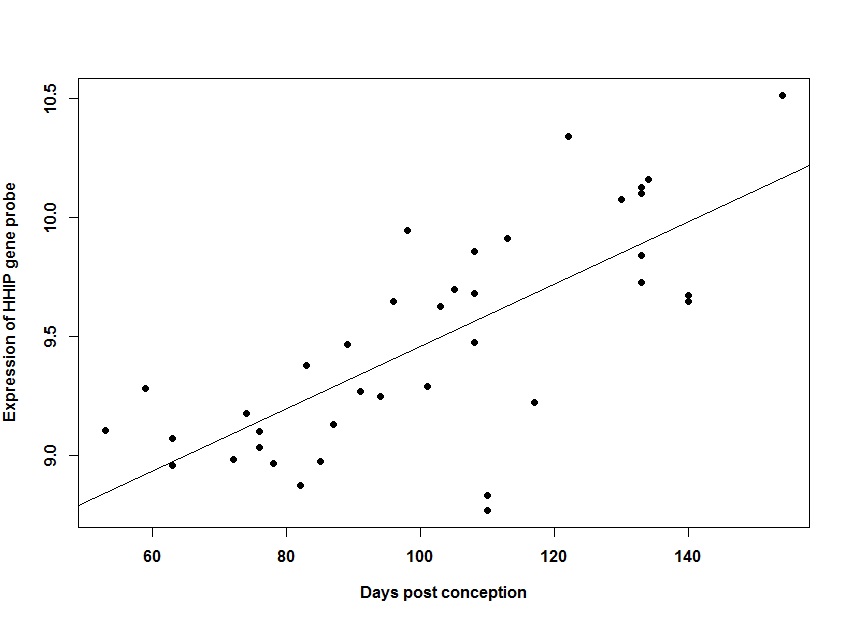

Supplement: Additional file 6: Figures S3. — HHIP mRNA expression in human lung across Pseudoglandular and Canalicular stages of development. Expression of HHIP gene probe 209815_at showed an increase in mRNA expression with increasing fetal lung age. (JPG 43 kb) [file 12931_2016_410_MOESM6_ESM.jpg]

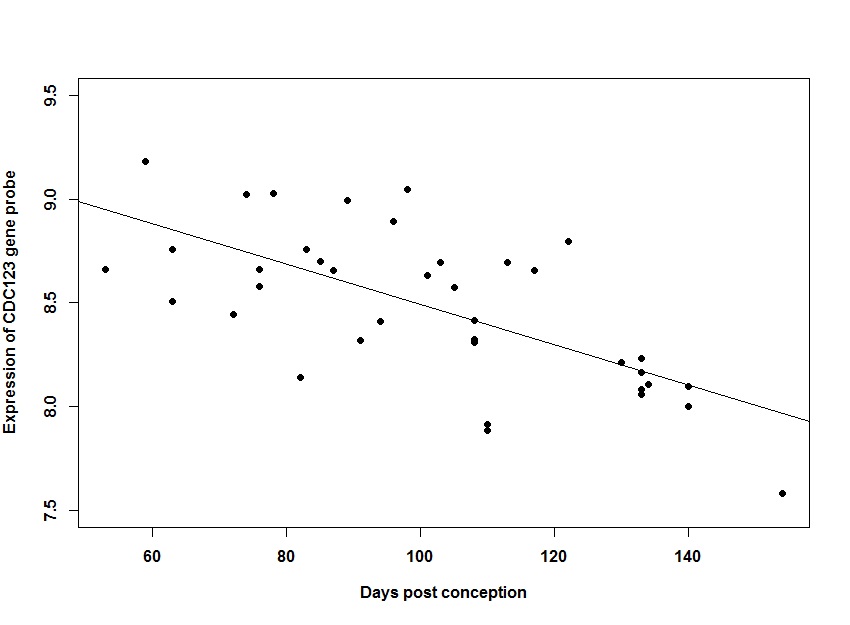

Supplement: Additional file 7: Figures S4. — CDC123 mRNA expression in human lung across Pseudoglandular and Canalicular stages of development. Expression of CDC123 gene probe 223503_at showed a decrease in mRNA expression with increasing fetal lung age. (JPG 43 kb) [file 12931_2016_410_MOESM7_ESM.jpg]

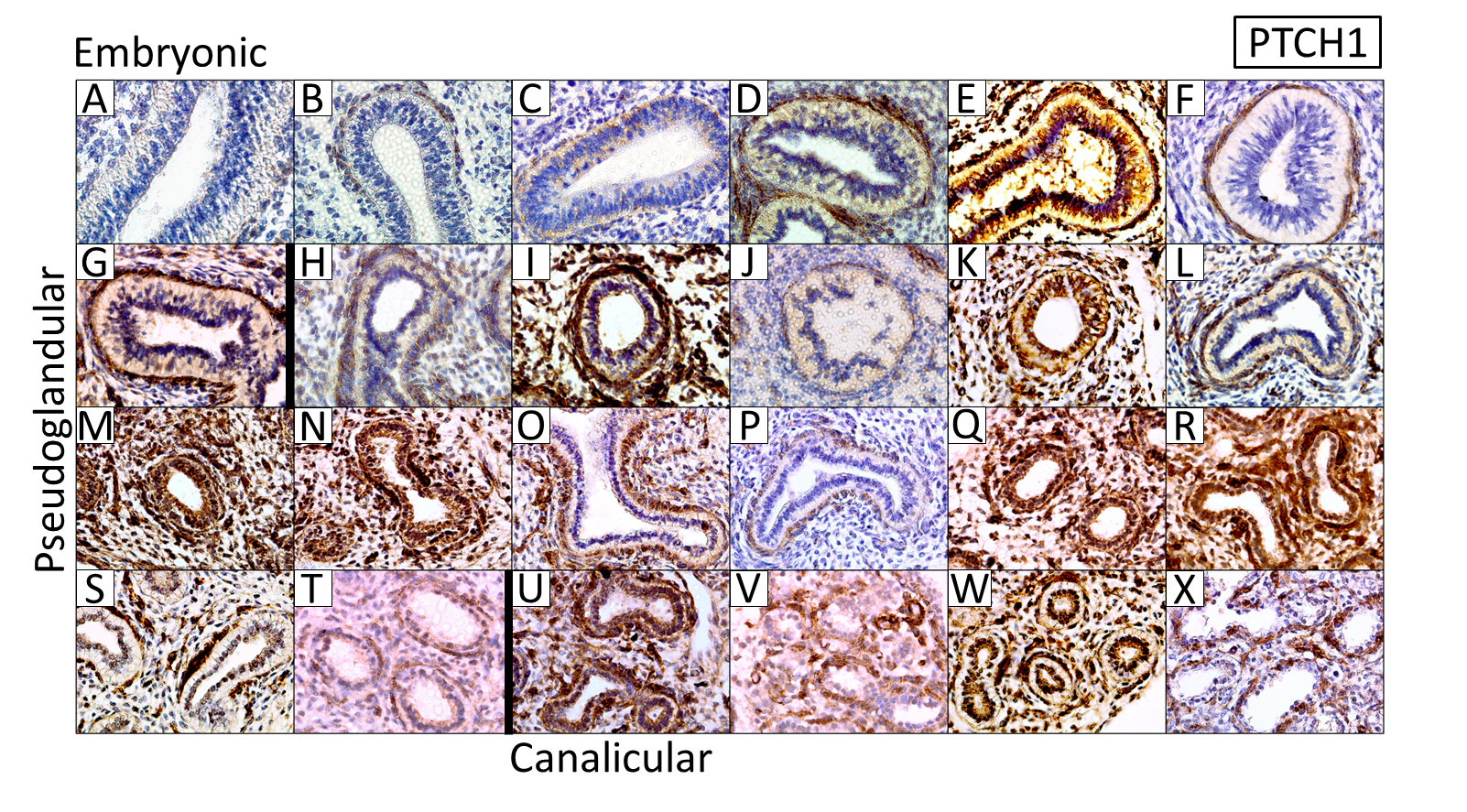

Supplement: Additional file 10: Figures S5. — Immunohistochemistry for PTCH1 in 24 fetal lung samples. The majority of fetal lungs showed strong or moderate immunopositivity for the PTCH1 protein. (A–G) embryonic stage, (H–T) Pseudoglandular stage and (U–X) Canalicular stage. An isotype control (not shown) gave no background staining. x40 Magnification. (TIF 3594 kb) [file 12931_2016_410_MOESM10_ESM.tif]

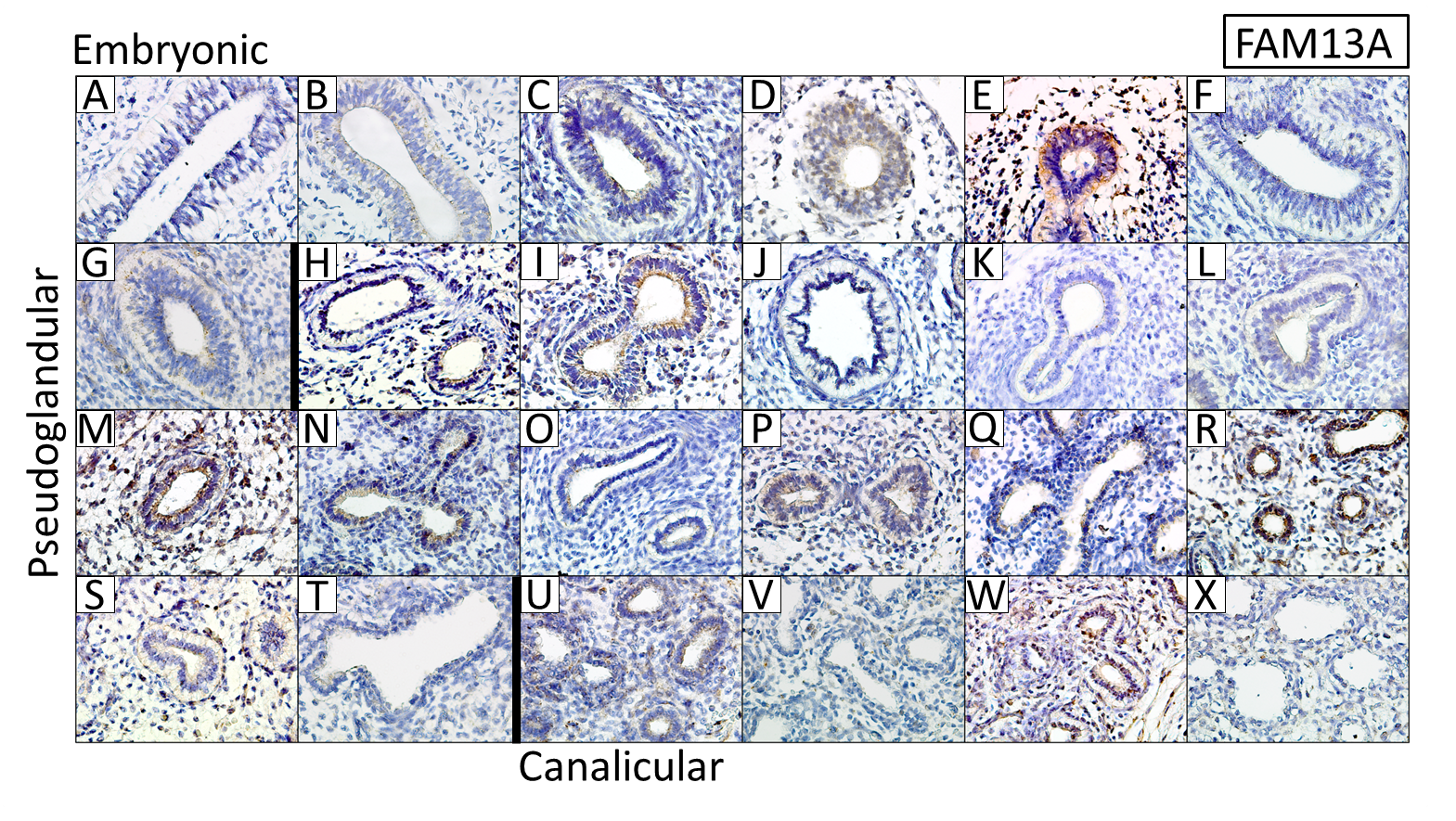

Supplement: Additional file 11: Figures S6. — Immunohistochemistry for FAM13A in 24 fetal lung samples. Fetal lung samples showed either low level or negative protein expression for FAM13A. (A–G) embryonic stage, (H–T) Pseudoglandular stage and (U–X) Canalicular stage. An isotype control (not shown) gave no background staining. x40 Magnification. (TIF 3390 kb) [file 12931_2016_410_MOESM11_ESM.tif]

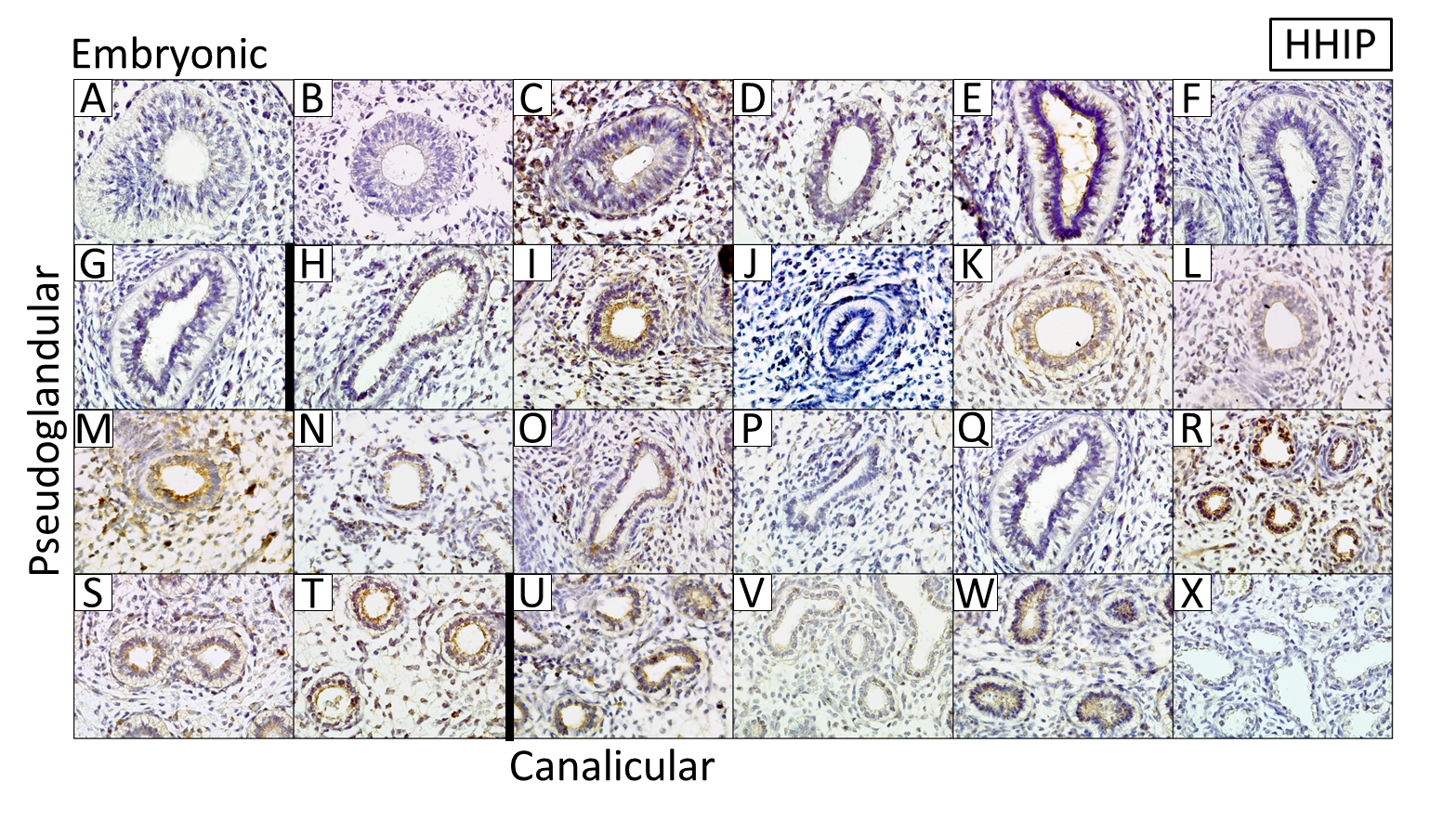

Supplement: Additional file 12: Figures S7. — Immunohistochemistry for HHIP in 24 fetal lung samples. HHIP protein expression was either not present or moderate throughout lung development. (A–G) embryonic stage, (H–T) Pseudoglandular stage and (U–X) Canalicular stage. An isotype control (not shown) gave no background staining. x40 Magnification. (TIF 3261 kb) [file 12931_2016_410_MOESM12_ESM.tif]
