# Supplementary material for: Genes associated with polymorphic variants predicting lung function are differentially expressed during human lung development
Source: Respir Res. 2016 Jul 29;17:95. doi: 10.1186/s12931-016-0410-z (PMC4966770; doi:10.1186/s12931-016-0410-z)
Supplement: Additional file 4: Table S3. — Lung function associated genes with significantly different expression during lung development. Affymetrix U133 Plus 2 array probe ID. Beta coefficient corresponds to the mean change in gene expression per day during the studied period (7-22 weeks of gestational age). (DOCX 22 kb) [file 12931_2016_410_MOESM4_ESM.docx]

**Table S3:** Lung function associated genes with different expression during lung development

| Gene | Probe | Adj P Value | Beta coefficient |
| --- | --- | --- | --- |
| *TMEM163* | 223503_at | 4.23E-09 | 0.0328382 |
| *TMEM163* | 1552626_a_at | 1.71E-08 | 0.0219712 |
| *PTCH1* | 209815_at | 2.96E-06 | -0.0123042 |
| *NPNT* | 225911_at | 6.65E-06 | 0.0113676 |
| *PRDM11* | 229687_s_at | 6.69E-06 | -0.0145780 |
| *BCL2* | 207004_at | 3.03E-05 | -0.0078140 |
| *HHIP* | 1556037_s_at | 3.23E-05 | 0.0138319 |
| *CDC123* | 223100_s_at | 5.72E-05 | -0.0090160 |
| *CDC123* | 201725_at | 5.97E-05 | -0.0096350 |
| *AGER* | 210081_at | 0.000190658 | 0.0150551 |
| *ARMC2* | 223866_at | 0.000269519 | 0.0065777 |
| *TSPYL4* | 212928_at | 0.000347776 | -0.0074899 |
| *TNS1* | 221246_x_at | 0.000416181 | 0.0065108 |
| *GSTCD* | 235387_at | 0.000854475 | -0.0115404 |
| *MTHFD1L* | 231094_s_at | 0.001160078 | -0.0051681 |
| *AIF1* | 207823_s_at | 0.001241938 | 0.0071076 |
| *RARB* | 211987_at | 0.001409069 | -0.0044030 |
| *NOTCH4* | 205247_at | 0.001576566 | 0.0077077 |
| *WWOX* | 223868_s_at | 0.002401611 | -0.0082566 |
| *HHIP* | 237466_s_at | 0.003507956 | 0.0119547 |
| *BCL2* | 203684_s_at | 0.004873179 | -0.0042379 |
| *MECOM* | 208434_at | 0.005271476 | 0.0057671 |
| *PRDM11* | 229688_at | 0.005538911 | -0.0076778 |
| *FAM13A* | 202973_x_at | 0.006295414 | 0.0092317 |
| *ADAM19* | 234208_at | 0.007438651 | 0.0057287 |
| *FAM13A* | 243020_at | 0.007503649 | 0.0117218 |
| *ADAM19* | 209765_at | 0.008108532 | 0.0055271 |
| *DAAM2* | 212793_at | 0.008115194 | -0.0076113 |
| *PRDM11* | 233067_at | 0.009000312 | -0.0056883 |
| *MTHFD1L* | 225520_at | 0.009522514 | -0.0103665 |
| *FAM13A* | 217047_s_at | 0.009933353 | 0.0087924 |
| *BMP6* | 215042_at | 0.011008411 | 0.0051900 |
| *PTCH1* | 208522_s_at | 0.011420378 | -0.0053706 |
| *THSD4* | 222835_at | 0.011749569 | -0.0074463 |
| *PDE4D* | 236610_at | 0.011938908 | 0.0104328 |
| *HTR4* | 207577_at | 0.012052715 | 0.0023823 |
| *RARB* | 217020_at | 0.012963281 | 0.0042669 |
| *FLJ20184* | 237128_at | 0.013303066 | 0.0059794 |
| *C10orf11* | 223703_at | 0.013626379 | 0.0080542 |
| *C10orf11* | 240772_at | 0.013631684 | 0.0134878 |
| *GSTCD* | 1554518_at | 0.014992322 | -0.0054719 |
| *ESR1* | 207672_at | 0.016749252 | 0.0029521 |
| *RARB* | 208412_s_at | 0.016874583 | -0.0055578 |
| *GPR126* | 1553025_at | 0.016893976 | 0.0041467 |
| *PTCH1* | 209816_at | 0.017182295 | -0.0109530 |
| *PDE4D* | 210837_s_at | 0.019211068 | 0.0061825 |
| *ESR1* | 217190_x_at | 0.021942159 | -0.0045759 |
| *PDE4D* | 204491_at | 0.023941030 | 0.0052592 |
| *AGER* | 217046_s_at | 0.025146987 | 0.0065857 |
| *FLJ20184* | 221119_at | 0.026302595 | 0.0042076 |
| *FAM13A* | 232628_at | 0.027001277 | 0.0154821 |
| *MECOM* | 237269_at | 0.027677807 | 0.0084431 |
| *RARB* | 208530_s_at | 0.032189828 | -0.0068868 |
| *PDE4D* | 210836_x_at | 0.045203570 | 0.0045935 |

Legend: Probe = Affymetrix U133 Plus 2 array probe ID. Adj P Value = Adjusted p-value (B-H method) for differential expression over time. The Beta coefficient corresponds to the mean change in gene expression per day during the studied period (7-22 weeks of gestational age) and shows the direction of effect. *selection based on adjusted p value <0.05.
